# Common impairments of emotional facial expression recognition in schizophrenia across French and Japanese cultures

**DOI:** 10.3389/fpsyg.2015.01018

**Published:** 2015-07-23

**Authors:** Takashi Okada, Yasutaka Kubota, Wataru Sato, Toshiya Murai, Fréderic Pellion, Françoise Gorog

**Affiliations:** ^1^Department of Psychiatry, Nagoya University Graduate School of MedicineAichi, Japan; ^2^Kouai HospitalOsaka, Japan; ^3^Health and Medical Service Center, Shiga UniversityShiga, Japan; ^4^Centre Hospitalier Sainte AnneParis, France; ^5^Primate Research Institute, Kyoto UniversityAichi, Japan; ^6^Department of Psychiatry, Kyoto University Graduate School of MedicineKyoto, Japan

**Keywords:** cross-culture, emotional facial expression, France, Japan, schizophrenia

## Abstract

To address whether the recognition of emotional facial expressions is impaired in schizophrenia across different cultures, patients with schizophrenia and age-matched normal controls in France and Japan were tested with a labeling task of emotional facial expressions and a matching task of unfamiliar faces. Schizophrenia patients in both France and Japan were less accurate in labeling fearful facial expressions. There was no correlation between the scores of facial emotion labeling and face matching. These results suggest that the impaired recognition of emotional facial expressions in schizophrenia is common across different cultures.

## Introduction

Individuals with schizophrenia are characterized by impairments in emotional responses to other individuals (World Health Organization, 1993). One of the most evident features of their emotional impairments is that individuals with schizophrenia have been shown to be impaired in recognizing emotions from facial expressions (e.g., Mandal et al., 1998; for a review, see Edwards et al., 2002).

Although a culturally invariant nature has been assumed for schizophrenia in the standardized diagnosis system (World Health Organization, 1993), evidence suggests a socio-cultural influence on the symptoms of this disease (cf. Thakker and Ward, 1998). It is unclear whether impaired facial expression processing in schizophrenia is culturally common or different across cultures. To clarify this issue, cross-cultural research is informative. However, almost all previous studies that assessed profiles of impaired recognition of emotional facial expressions in schizophrenia solely targeted western participants using western models as stimuli. To date, only one study has explored the nature of facial expression recognition impairment in schizophrenia across western and eastern cultures (Habel et al., 2000). The researchers tested American, German, and Indian patients with schizophrenia. The schizophrenia patients in all groups showed impaired recognition of emotional facial expressions, suggesting a common nature of the impairment. However, the researchers only tested western models using two emotional expressions, happy and sad, and neutral expressions. This study of facial expression also did not test basic face perception abilities. Therefore, the cross-cultural and cross-emotional nature of impaired recognition of emotional facial expressions in schizophrenia and the relationship to basic face perception ability remain inconclusive.

Another concern about previous studies of emotional facial misrecognition in schizophrenia is that individuals with schizophrenia have many perceptual and cognitive impairments (Chapman and Chapman, 1973, 1978), including deficits in face perception (e.g., Borod et al., 1993). It has been debated whether the impairments in facial affect recognition may just reflect dysfunctions in emotional expression processing. Some studies showed that impaired recognition of emotional facial expressions in schizophrenia was a result of basic impairments in face perception (e.g., Kerr and Neale, 1993; Salem et al., 1996), but others found that patients with schizophrenia were impaired in recognizing emotional facial expressions independently from their problems of basic perception (e.g., Novic et al., 1984). It therefore remains unsolved whether the misrecognition of emotional facial expression is independent from other cognitive dysfunctions.

Here, we explored the impaired recognition of emotional facial expressions in schizophrenia across western and eastern cultures. We tested patients with schizophrenia and age-matched normal controls in France and Japan. To test facial expression recognition, we presented facial expressions of Caucasian and Japanese models who showed six basic emotions (cf. Ekman and Friesen, 1975) and asked the participants to label them. To test the non-emotional face perception abilities, we investigated the matching of unfamiliar faces. Based on previous evidence (Habel et al., 2000), we predicted that the schizophrenic patients would show lower accuracy in labeling emotional facial expressions across French and Japanese cultures and the impairment would be independent from their face-perception ability.

## Materials and Methods

### Participants

The schizophrenia group comprised 26 patients; 14 French (seven females, seven males; age, *M* = 30.7, *SD* = 8.1; years from onset, *M* = 8.9, *SD* = 6.1) and 12 Japanese (six females, six males; age, *M* = 34.3, *SD* = 10.3; years from onset, *M* = 9.2, *SD* = 9.7). The patients were diagnosed as Paranoid Schizophrenia (F20.0) according to ICD-10 (World Health Organization, 1993).

The control group comprised 24 healthy adult volunteers; 12 French (six females, six males; age, *M* = 34.7, *SD* = 9.0) and 12 Japanese (six females, six males; age, *M* = 34.3, *SD* = 3.8), who were age-matched with the schizophrenia group. All participants were right-handed. The schizophrenia patients were remitted and stable during the test. There was no history of substance abuse, head injury, or neurological disease.

All participants gave informed consent to participate in this study. This study was approved by the ethics committees of Kouai Hospital and Centre Hospitalier Sainte Anne and conducted in accordance the Declaration of Helsinki.

### Procedures

#### Expression Recognition Task

A total of 48 photographs of facial expressions depicting six basic emotions (anger, disgust, fear, happiness, sadness, and surprise) were used as stimuli. Half of these photographs were pictures of Caucasian models and the remaining half were pictures of Japanese models. These pictures were chosen from standardized photograph sets (Ekman and Friesen, 1976; Matsumoto and Ekman, 1988). The events were controlled by SuperLab Pro 2.0 (Cedrus, San Pedro, CA, USA) implemented on a Windows computer.

A label-matching paradigm previously used by Sato et al. (2002) was employed to assess participants’ recognition of emotional facial expressions as in a previous study (Kubota et al., 2003). Pictures of people whose faces expressed various emotions were presented on the monitor one at a time in a random order. Verbal labels identifying the six basic emotions were presented next to each photograph (Japanese labels for Japanese participants and French labels for French participants). Participants were asked to select the label that best described the emotion shown in each photograph. They were instructed to consider all six alternatives carefully before responding. No time limits were set, and no feedback was provided about performance. Participants saw photos depicting six emotional expressions of eight individuals (two Japanese males, two Japanese females, two Caucasian males, and two Caucasian females), resulting in a total of 48 trials for each participant. A break was given after every 12 photos. The order of the photos and labels shown to the participant was randomized.

Before starting the task, the participant was briefed on the content of the task and the way to answer the questions. They were asked to give a short description of each label in order to make sure that they understood the meaning of the labels. All participants confirmed they knew the meanings of all six labels of basic emotions. The participant started the task after a few examples and five practice trials.

#### Face Perception Task

The shortened version (13 items) of the Benton Facial Recognition Test (Benton et al., 1978) was conducted as in previous studies (e.g., Kubota et al., 2003). Performance on this test is based on perceptual factors and reflects basic visual face-processing mechanisms (e.g., Bentin et al., 1999). Caucasian models were used for all of the face stimuli. Participants were required to match a target face with one picture or with up to three pictures of the same person presented in a six stimulus-array of faces. No time limits were set, and no feedback was provided regarding performance.

### Data Analysis

Analyses were conducted with SPSS 10.0J (SPSS, Tokyo, Japan). Correct response percentages for the expression recognition task were subjected to a four-way analysis of variance (ANOVA) with diagnostic group (schizophrenia or control) and participant culture (French or Japanese) as the between-participants factors and emotion (anger, disgust, fear, happiness, sadness, or surprise) and stimulus culture (Caucasian or Japanese) as the within-participants factors. The total correct response number for the face perception task was analyzed by a two-way ANOVA with diagnostic group (schizophrenia or control) and participant culture (French or Japanese) as the between-participants factors. In cases where the assumption of sphericity was not met (Mauchley’s sphericity test, *p* < 0.05), the Greenhouse–Geisser adjusted degree of freedom was used. Follow-up analyses were conducted using simple effects analyses [i.e., examining main effects of one factor separately at each level of the other factors for two-way (simple main effect) or three-way (simple-simple main effect) interactions; Kirk, 1995; Tabachnick and Fidell, 2001] and multiple comparisons using Ryan’s method. Because preliminary analysis showed no significant effects of participants’ sexes, the scores of both sex groups were assessed together. Pearson correlations were also calculated between the performance of expression recognition of emotional category and that of face perception in each group (French schizophrenia, Japanese schizophrenia, French control, and Japanese control). Although our preliminary analysis for the data distribution showed that the data for happy and surprised expressions of all diagnostic group, participant culture, and stimulus culture conditions were not normally distributed (Kolmogorov–Smirnov test, *p* < 0.05), we report the aforementioned analyses including these data, because (1) ANOVAs with more than 20 degrees of freedom are robust for the violation of normality of sampling distributions (Tabachnick and Fidell, 2001), (2) the ANOVAs with and without these data showed the same patterns of significant results, and (3) parametric and non-parametric correlations showed the same patterns of significant results. Significance level was set at *p* < 0.05.

## Results

### Expression Recognition Task

**FIGURE 1:**
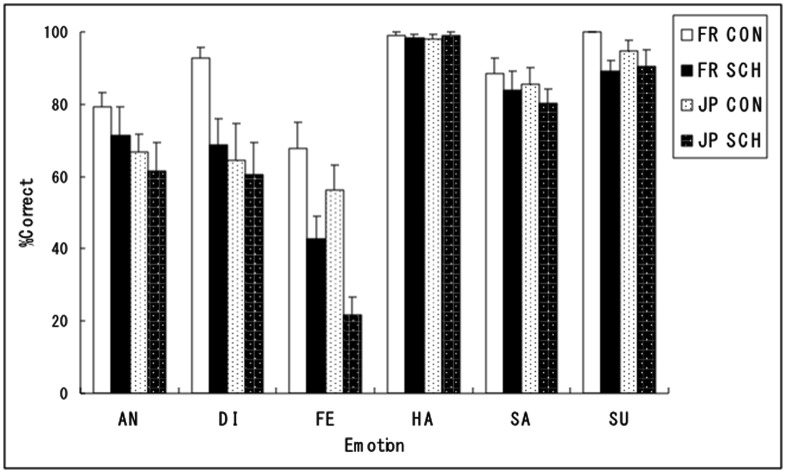
**Mean (with standard error) percentages of correct responses of the emotional expression recognition task in the normal control (CON) and schizophrenia (SCH) groups in French (FR) and Japanese (JP) cultures.** AN = anger; DI = disgust; FE = fear; HA = happiness; SA = sadness; SU = surprise.

For the correct response percentages for the expression recognition task (**Figure 1**; Supplementary Table S1), a four-way ANOVA with diagnostic group, participant culture, emotion, and stimulus culture as factors was conducted. The results showed a significant interaction between diagnostic group and emotion [*F*(5,230) = 4.11, *p* < 0.005, $\eta_{p}^{2}$ = 0.089], indicating that diagnostic groups differed in facial expression recognition differently across emotions. The main effect of diagnostic group was also significant [*F*(1,46) = 14.45, *p* < 0.001, $\eta_{p}^{2}$ = 0.264], indicating that the overall accuracy of facial affect recognition was lower in the schizophrenia group than the control group. There were also main effects of emotion [*F*(5,230) = 53.03, *p* < 0.001, $\eta_{p}^{2}$ = 0.525], showing the order of accurate recognition for happiness = surprise > sadness > disgust > anger > fear, and participant culture [*F*(1,46) = 9.74, *p* < 0.005, $\eta_{p}^{2}$ = 0.145], indicating higher facial expression recognition in the French than Japanese group. There was also a marginally significant participant culture × emotion × stimulus culture interaction [*F*(5,230) = 2.15, *p* < 0.1, $\eta_{p}^{2}$ = 0.043], indicating a trend that French participants were more accurate in the recognition of Caucasian models’ disgusted expressions. No other main effects or interactions were significant (*p* > 0.1), including the main effect of stimulus culture [*F*(1,46) = 0.90, $\eta_{p}^{2}$ = 0.018], diagnostic group × participant culture interaction [*F*(1,46) = 0.05, $\eta_{p}^{2}$ = 0.001], diagnostic group × stimulus culture interaction [*F*(1,46) = 1.68, $\eta_{p}^{2}$ = 0.034], participant culture × emotion interaction [*F*(5,230) = 1.85, $\eta_{p}^{2}$ = 0.037], participant culture × stimulus culture interaction [*F*(1,46) = 0.32, $\eta_{p}^{2}$ = 0.007], emotion × stimulus culture interaction [*F*(5,230) = 1.77, $\eta_{p}^{2}$ = 0.035], diagnostic group × participant culture × emotion interaction [*F*(5,230) = 1.30, $\eta_{p}^{2}$ = 0.026], diagnostic group × emotion × stimulus culture interaction [*F*(5,230) = 0.74, $\eta_{p}^{2}$ = 0.015], diagnostic group × participant culture × stimulus culture interaction [*F*(1,46) = 0.00, $\eta_{p}^{2}$ = 0.000], and diagnostic group × participant culture × emotion × stimulus culture interaction [*F*(5,230) = 0.60, $\eta_{p}^{2}$ = 0.012].

For follow-up analyses of the diagnostic group × emotion interaction, the simple main effect of diagnostic group was analyzed in each emotion category. The results revealed significant simple main effects of diagnostic group for the expressions of fear [*F*(1,276) = 29.53, *p* < 0.001] and disgust [*F*(1,276) = 6.66, *p* < 0.05], indicating that the schizophrenia group was less accurate than the control group in the recognition of emotional expressions of fear and disgust. There was no other simple main effect of diagnostic group (*p* > 0.1).

To further confirm the common impairments in facial expression recognition in schizophrenia across French and Japanese cultures, the simple-simple main effect of diagnostic group in each participant culture in each emotion category was analyzed. For fear, the effect was significant in both the French [*F*(1,288) = 10.46, *p* < 0.005] and Japanese [*F*(1,288) = 26.32, *p* < 0.001] groups, indicating that both French and Japanese schizophrenia groups were less accurate relative to their control groups in the recognition of fearful emotional expressions. For disgust, the simple-simple main effect of diagnostic group was significant in the French [*F*(1,288) = 9.72, *p* < 0.005] but not in the Japanese [*F*(1,288) = 0.25, *p* > 0.1] participants. No other simple-simple main effects of diagnostic group were significant (*p* > 0.1). Because the result of disgusted expression recognition was not clear-cut, we only discuss the results of fearful expression recognition.

### Face Perception Task

**FIGURE 2:**
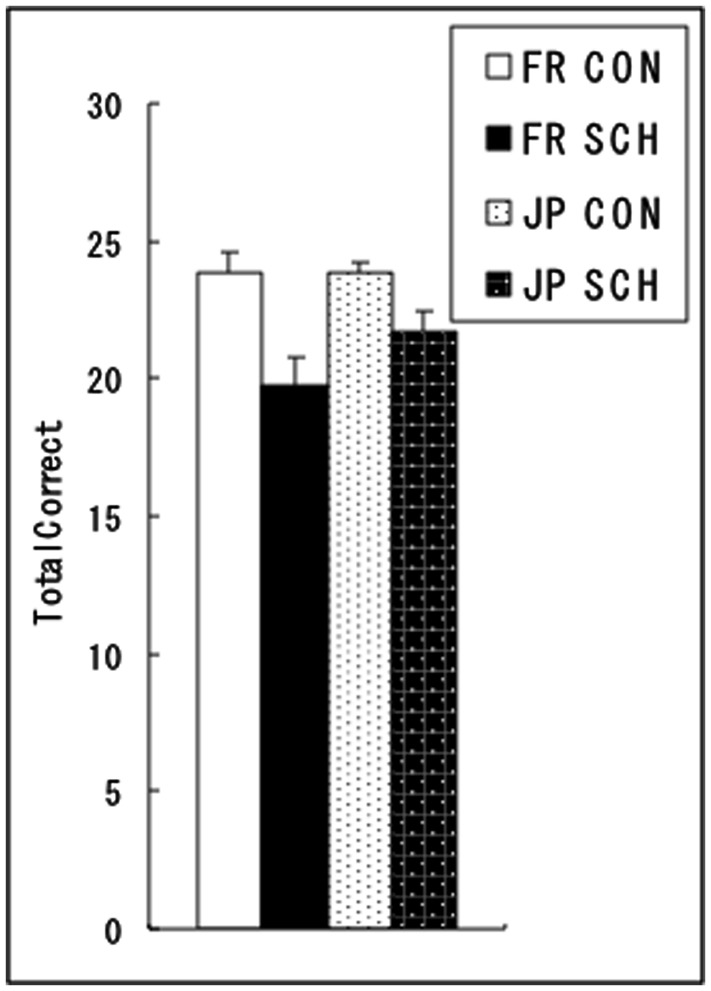
**Mean (with standard error) total numbers of correct responses of the face perception task in the normal control (CON) and schizophrenia (SCH) groups in French (FR) and Japanese (JP) cultures**.

On the face perception task (**Figure 2**), a two-way ANOVA with diagnostic group and participant culture as factors revealed a significant main effect of diagnostic group [*F*(1,46) = 15.96, *p* < 0.001] on the total number correct, indicating that the schizophrenia group scored lower in the perception of faces. There were no other significant main effects or interactions (*p* > 0.1).

### Relationship between Expression Recognition and Face Perception

To examine the possibility that impaired recognition of emotional facial expression could result from basic face perception deficits rather than specific emotional processing difficulties, the correlation between the performance of emotion recognition of each emotional category and that of face perception in each group was calculated (**Table 1**). There were significant correlations only in the case of angry expressions in Japanese and French schizophrenia groups (*r* > 0.59, *p* < 0.05). There was no other significant correlation on other emotional categories or in other groups (*p* > 0.1).

**Table 1:** Correlations between the performances of facial expression recognition and face perception.

		Anger	Disgust	Fear	Happiness	Sadness	Surprise
France	Control	-0.28	0.34	0.19	0.34	0.30	-^A^
	Schizophrenia	0.52^∗∗^	0.39	0.05	-0.14	0.32	0.16
Japan	Control	-0.56^+^	0.12	-0.10	-0.22	-0.17	-0.48
	Schizophrenia	0.57^∗^	0.17	0.38	-0.05	0.30	0.13

*^∗^*p* < 0.05; ^∗∗^*p* < 0.01; ^+^*p* < 0.1.*

*^A^No data because of no variance.*

## Discussion

Our results showed that both schizophrenia groups were impaired in the recognition of fearful facial expressions. This deficit for the recognition of negative emotions, such as fear, in the schizophrenia groups is consistent with previous findings (Garfields et al., 1987; Mandal et al., 1998; Edwards et al., 2001; Kubota et al., 2003).

More importantly, our results showed that the impaired recognition of emotional facial expressions was common across French and Japanese cultures. Both French and Japanese schizophrenia groups showed lower performance in the recognition of fearful facial expressions than did the control groups. There was no effect related to the cultures of the stimulus models. These results are consistent with those in a previous study that investigated American, German, and Indian individuals with schizophrenia, and reported common impairments in the recognition of emotional facial expression in schizophrenia (Habel et al., 2000). However, as described in Introduction, that previous study investigated only two emotions and only the stimuli of western models. Our results extend the previous finding and suggest that the impairment in recognizing fearful facial expressions in schizophrenia is common across western and eastern cultures.

Our results also showed that, although both French and Japanese schizophrenia groups showed lower accuracy in the face perception task, these basic perceptual deficits were not correlated with impaired facial affect recognition. There remain debates whether the impairments in facial affect recognition in schizophrenia might reflect the dysfunctions in other basic cognitive abilities (e.g., Salem et al., 1996). These results are consistent with findings from other neuropsychiatric disorders such as autism spectrum disorders (ASDs). Hefter et al. (2005) and Uono et al. (2011) reported that the deficits in the facial expression recognition and face perception did not correlate in individuals with ASD. Together with these data, our results suggest that the common impairments in recognizing fearful facial expressions in schizophrenia across Japanese and French cultures are independent from the deficit in basic face perception.

Besides the effect of diagnostic group, our results showed that the French participants were more accurate in identifying emotional facial expressions than Japanese participants. This is in line with previous reports and Matsumoto (1989, 1992) argued that members of collectivistic cultures such as the Japanese may demonstrate decreased emotion recognition when judging stimuli from other regions, particularly negative emotions. Our results also showed a trend that French participants more accurately recognized Caucasian models’ disgusted expressions. The result is consistent with an in-group advantage such that emotions are recognized more accurately when expressed by members of the same cultural group (for review, see Elfenbein and Ambady, 2002). These possible cultural effects on emotional recognition performance were observed across both of our patient and control groups, but seemed to have no significant consequences on the observed fear recognition impairments in schizophrenia.

The limitations of this study should be acknowledged. The sample size was small and only French and Japanese cultures were tested. Generalization to other western or eastern cultures remains unproven. Further investigation of individuals with schizophrenia from other cultural backgrounds using the present paradigm are warranted.

## Role of Funding Source

Funding for this study was provided by research grants from the Ministry of Education, Culture, Sports, Science and Technology of Japan (23791327) for Takashi Okada, and the funds from the Japan Society for the Promotion of Science Funding Program for Next Generation World-Leading Researchers (LZ008) for Wataru Sato. The funders had no role in study design, data collection and analysis, decision to publish, or preparation of the manuscript.

## Author Contributions

All authors designed the study. Data collection was performed by TO and YK. Statistical analysis was performed by TO and WS. All authors wrote and have approved the manuscript.

## Conflict of Interest Statement

Funding for this study was provided by research grants from the Ministry of Education, Culture, Sports, Science and Technology of Japan (23791327) for Takashi Okada, and the funds from the Japan Society for the Promotion of Science Funding Program for Next Generation World-Leading Researchers (LZ008) for Wataru Sato. The funders had no role in study design, data collection and analysis, decision to publish, or preparation of the manuscript.
