# Supplementary material for: Common impairments of emotional facial expression recognition in schizophrenia across French and Japanese cultures
Source: Front Psychol. 2015 Jul 23;6:1018. doi: 10.3389/fpsyg.2015.01018 (PMC4511826; doi:10.3389/fpsyg.2015.01018)
Supplement: Supplementary file 1 [file Table_1.DOCX]

**Supplementary Table 1.**

Mean (with standard error) percentages of correct responses of the emotional expression recognition task.

| Participant | | Stimulus | |  |  |  |  |  |  |  |  |  |  |  |
| --- | --- | --- | --- | --- | --- | --- | --- | --- | --- | --- | --- | --- | --- | --- |
|  |  | Caucasian | |  |  |  |  |  | Japanese | |  |  |  |  |
| Culture | Diagnosis | AN | DI | FE | HA | SA | SU |  | AN | DI | FE | HA | SA | SU |
| French | Control | 79.2 | 97.9 | 72.9 | 97.9 | 87.5 | 100.0 |  | 79.2 | 87.5 | 62.5 | 100.0 | 89.6 | 100.0 |
|  |  | (6.0) | (2.1) | (7.2) | (2.1) | (4.9) | (0.0) |  | (6.0) | (4.9) | (8.4) | (0.0) | (7.2) | (0.0) |
|  | Schizophrenia | 69.2 | 67.3 | 67.3 | 96.2 | 96.2 | 94.2 |  | 69.2 | 67.3 | 51.9 | 100.0 | 76.9 | 96.2 |
|  |  | (6.4) | (10.4) | (7.7) | (2.6) | (2.6) | (4.2) |  | (8.1) | (10.4) | (8.2) | (0.0) | (7.2) | (3.8) |
| Japanese | Control | 67.9 | 75.0 | 41.1 | 98.2 | 82.1 | 85.7 |  | 75.0 | 62.5 | 44.6 | 98.2 | 85.7 | 92.9 |
|  |  | (7.6) | (7.9) | (7.2) | (1.8) | (5.5) | (5.1) |  | (9.8) | (7.8) | (7.5) | (1.8) | (5.7) | (3.1) |
|  | Schizophrenia | 61.5 | 61.5 | 23.1 | 98.1 | 78.8 | 88.5 |  | 55.8 | 65.4 | 17.3 | 100.0 | 76.9 | 94.2 |
|  |  | (8.8) | (10.1) | (6.0) | (1.9) | (8.4) | (6.1) |  | (8.6) | (10.4) | (6.6) | (0.0) | (4.4) | (3.0) |
| AN = anger; DI = disgust; FE = fear; HA = happiness; SA = sadness; SU = surprise. | | | | | | | | | |  |  |  |  |  |
